# Terrestrial chemical cues help coral reef fish larvae locate settlement habitat surrounding islands

**DOI:** 10.1002/ece3.53

**Published:** 2011-12

**Authors:** Danielle L Dixson, Geoffrey P Jones, Philip L Munday, Morgan S Pratchett, Maya Srinivasan, Serge Planes, Simon R Thorrold

**Affiliations:** 1 School of Marine and Tropical Biology, James Cook UniversityTownsville, Queensland, Australia; 2 ARC Centre of Excellence for Coral Reef Studies, James Cook UniversityTownsville, Queensland, Australia; 3 Laboratoire Ècosystèmes Aquatiques Tropicaux et Mèditerranèens, UMR 5244 CNRS-EPHE-UPVD, Universitè de PerpignanPerpignan Cedex, France; 4 Biology Department MS Number 50, Woods Hole Oceanographic InstitutionWoods Hole, MA

**Keywords:** Chemical ecology, Dispersal, Habitat selection, Olfactory cues, Recruitment, Settlement site selection

## Abstract

Understanding the degree of connectivity between coastal and island landscapes and nearby coral reefs is vital to the integrated management of terrestrial and marine environments in the tropics. Coral reef fish are capable of navigating appropriate settlement habitats following their pelagic larval phase, but the mechanisms by which they do this are unclear. The importance of olfactory cues in settlement site selection has been demonstrated, and there is increasing evidence that chemical cues from terrestrial sources may be important for some species. Here, we test the olfactory preferences of eight island-associated coral reef fish recruits and one generalist species to discern the capacity for terrestrial cue recognition that may aid in settlement site selection. A series of pairwise choice experiments were used to evaluate the potential role that terrestrial, water-borne olfactory cues play in island–reef recognition. Olfactory stimuli tested included near-shore water, terrestrial rainforest leaf litter, and olfactory cues collected from different reef types (reefs surrounding vegetated islands, and reefs with no islands present). All eight island-associated species demonstrated high levels of olfactory discrimination and responded positively toward olfactory cues indicating the presence of a vegetated island. We hypothesize that although these fish use a suite of cues for settlement site recognition, one mechanism in locating their island/reef habitat is through the olfactory cues produced by vegetated islands. This research highlights the role terrestrial olfactory cues play in large-scale settlement site selection and suggests a high degree of ecosystem connectivity.

## Introduction

The importance of integrated management of terrestrial and marine environments has been highlighted by numerous studies (Allison et al. 1998; Jameson et al. 2002; Aronson and Precht 2006; Dixson et al. 2008); however, reserve networks are often designed in either terrestrial or marine ecosystems, ignoring interactions between the two (Beck 2003). There are many examples of how one ecosystem can be jeopardized as a result of anthropogenic activities in another (Stoms et al. 2005). With nearly half of the world's population residing within 150 km of a coastline, the need for effective integrated management of coastal ecosystems is vital (Cohen et al. 1997). Coastal ecosystems, including terrestrial, freshwater, and marine environments are connected by the important exchange of materials, energy, and organisms (Reiners and Driese 2001; Stoms et al. 2005). For example, coastal mangroves have been identified as an important nursery habitat for many coral reef fish species, including species of commercial interest (Nagelkerken et al. 2000), however, 30–60% of the world's mangroves have already been lost from human development (Valiela et al. 2001; Upadhyay et al. 2002; Ellison 2008). At the same time, many coral reefs have been degraded by sedimentation and eutrophication from coastal development and agriculture, causing losses in diversity and abundance of reef species (McCulloch et al. 2003; Jones et al. 2004; Hughes et al. 2010).

The ability of dispersing individuals to locate suitable habitat is critical for their future survival and reproduction, which in turn influences the replenishment and persistence of adult populations (Rosenzweig 1981; Morris 2003). Most marine organisms begin life as pelagic larvae and, depending on the species, spend days to months in the pelagic environment. Regardless of larval duration, suitable adult habitat must be located at the conclusion of the larval stage. Settlement-stage larvae are thought to use a variety of settlement cues to locate suitable habitat, for example, chemical cues given off by living substrates (e.g., algae Steinberg and de Nyes 2002; Williamson et al. 2000; coral Ben–Tzvi et al. 2010) and conspecifics (e.g., barnacles Thiyagarajan 2010; fish Atema et al. 2002; Kingsford et al. 2002; Lecchini et al. 2005). Olfactory stimuli are recognized as an important cue for habitat location in diadromous organisms; with studies conducted on eels, lamprey, and fish identifying odor cues as important components for these migratory organisms (McCleave and Jellyman 2002; Hale et al. 2009; Vrieze et al. 2010). To date, most research on the chemical cues used by fish larvae for orientation and habitat selection has focused on chemicals produced by reef organisms. However, Dixson et al. (2008) showed that settlement-stage larvae of the anemonefish, *Amphiprion percula*, were positively attracted to the odor of terrestrial leaf litter, most likely because their symbiotic anemones are associated with reefs surrounding vegetated islands. The positive response of *A. percula* to the odor cues of terrestrial rainforest vegetation was found to be innate. It was suggested that the ability for terrestrial cues, such as leaf litter, to be exported away from island-based reefs allows detection at greater distances than traditional settlement cues such as anemone or coral odor.

Offshore islands are important ecosystems within the marine environment, often harboring a diversity of habitats and species, and are the focal point of human activity (Fernandes et al. 2005). Islands in remote locations typically display large numbers of endemic species (Jones et al. 2002; Allen 2007), as well as high levels of self-recruitment within populations (Almany et al. 2007). Consequently, mechanisms by which larvae locate suitable habitats are likely to be especially important to the persistence of such species. A better understanding of the mechanisms utilized by island-associated species at settlement will help to determine how populations on isolated islands are maintained. Understanding the degree to which the terrestrial landscape and the nearby coral reefs are connected is vital in achieving this goal.

The mechanisms that reef fish larvae use to find their way through the pelagic environment, and ultimately locate a suitable demersal settlement site, remain poorly understood (see Leis et al. 2011). Most fish larvae begin the pelagic stage with limited locomotory abilities and with incompletely developed sensory organs, however conclude the pelagic phase as competent swimmers (Stobutski and Bellwood 1998; Fisher and Bellwood 2002) with highly developed sensory systems (Kingsford et al. 2002). The physical capabilities larvae display during the latter portion of the larval period has led biologists to conclude that larval behavior influences settlement site selection at a variety of spatial scales (Atema et al. 1988; Kingsford et al. 2002; Montgomery et al. 2006; Leis 2007; Cowen and Sponaugle 2009). The larvae of coral reef fishes possess the necessary sensory morphology for detecting chemical cues and have been demonstrated to use chemical cues for orientation and habitat selection (Atema et al. 2002; Kingsford et al. 2002; Lechinni et al. 2005). It is thought that the primary sense used in detecting chemical cues is olfaction (Leis et al. 2011). Olfactory cues have been shown to play an important role for settling larval fish in the recognition of microhabitats (Sweatman 1983; Elliott et al. 1995; Arvedlund et al. 1999; Lechinni et al. 2005; Nangelkerken et al. 2008), food (Dempsey 1978; Knutsen 1992; Døving et al. 1994; Batty and Hoyt 1995; Kolkovski et al. 1997), conspecifics (Sweatman 1983, 1988; Ben–Tzvi et al. 2010), and predator avoidance (Dixson et al. 2010). Olfactory cues may also be important for orientation and navigation at much larger scales. For example, Gerlach et al. (2007) demonstrated that larvae are able to distinguish between reefs within a 10-km radius, indicating not only that olfactory cues may be useful over a greater distance than previously recognized but also that individual reefs have distinct characteristic odors that larvae are able to identify and respond to.

The aim of this study was to investigate the role that terrestrial olfactory cues play in settlement site selection among a variety of reef fishes associated with coral reefs adjacent to islands. Using similar methods as described in Dixson et al. (2008), we first examined the extent to which nine species from three reef fish families are restricted to the fringing reefs surrounding islands in Kimbe Bay, Papua New Guinea. We hypothesize that species with strong island–reef association will use cues produced by the terrestrial island mass for reef identification. Specifically, we predicted that olfactory cues are used to identify between island and nonisland reefs and that at least one of the olfactory cues utilized to distinguish between reef types is a product of the terrestrial system. A series of pairwise choice tests were conducted to test the ability of newly settled recruits to discriminate between different olfactory cues in the water column. Olfactory trials aimed to determine if newly settled recruits were able to distinguish between: (1) water taken from a reef surrounding an island compared to water from a reef where no island was present; (2) all combinations of water taken at different distances from the island source; and lastly (3) water treated with terrestrial rainforest vegetation compared to untreated offshore water. We hypothesize that island-associated juveniles will prefer island water compared to water from reefs without islands, water from near islands over offshore water, and water containing chemical cues from island vegetation over untreated water.

## Materials and Methods

### Study locations and species

**Figure 1:**
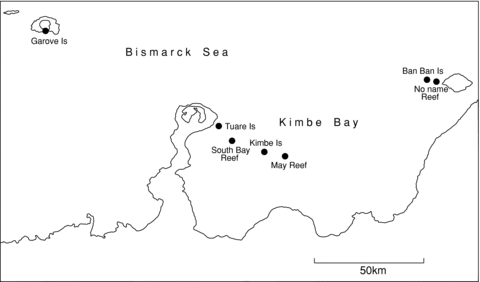
Map showing the location of Kimbe Bay (New Britain, Papua New Guinea) and the seven study location. Four locations were fringing reefs surrounding small islands covered with rainforest vegetation (Garove Island, Tuare Island, Kimbe Island, Ban Ban Island) and three were emergent reefs with no island (South Bay Reef, May Reef, and No Name Reef).

The study was carried out in Kimbe Bay (5°20′S, 150°15′E) on the island of New Britain, Papua New Guinea (Fig. 1). The study encompassed seven locations, including four reefs surrounding vegetated islands (Kimbe Island, Ban Ban Island, Garove Island, and Tuare Island) and three emergent reefs without islands (May Reef, South Bay Reef, and No name Reef).

Eight reef fish species, which displayed strong island association were chosen as study species, including: two species of butterflyfishes (Chaetodontidae) *Chaetodon vagabundus*, and *C. rafflesii*; four species of damselfishes (Pomacentridae), *A. percula*, *Pomacentrus simsiang*, *Dischistodus prosopotaenia*, and *Dascyllus melanurus*; and two species of wrasses (Labridae), *Halichoeres chloropterus*, and *H. argus*. *Amphiprion melanopus* (Pomacentridae), was also collected as a control. This species is found on both reefs surrounding island locations as well as reefs where no islands are present. Newly settled *A. melanopus* recruits were collected from the island locations to determine the reaction of a generalist fish to island-based cues. All study species were collected as settled recruits using the protocols described in Dixson et al. (2008), using minimal amount of clove oil and hand nets. After collection, fish were held individually in 1-L plastic bags for 2 h, following this recruits were tested for olfactory cue preferences within 24 h.

### Association with island reefs

To assess the importance of island-based chemical cues, island-associated coral reef fish recruits were needed. To estimate densities of the study species at island and nonisland locations, fish were surveyed along four randomly placed 50 × 4 m transects at each of six sites; three reefs not associated with islands (Margett's Reef, May Reef, and South Bay Reef) and three reefs that surround offshore islands (Kimbe Island, Tuare Island, Kapepa Island). Within each transect, the number of individuals of each fish species was recorded. Transects on reefs surrounding islands were placed 5 m from and parallel to the shoreline. On reefs with no islands, transects were placed 5 m from the leeward side of the reef. A nested analysis of variance (ANOVA) was used to compare the log-transformed transect data of fish densities between reefs with and without islands. This information ensures that species used associate strongly with the island habitat.

### General protocol for field olfactory choice trials

A two-channel choice flume (13 × 4 cm) developed by Gerlach et al. (2007) was used to assess the ability of newly settled fishes to discriminate between water containing different odor stimuli. This apparatus was designed to conduct pairwise choice experiments, with fish able to freely choose between water flowing from two different sources. Water from the two different sources was gravity fed into the choice flume that was partitioned along half of its length. Fish were released at the downstream end of the flume where they were free to move to either side or swim toward the preferred water source. Using the protocols outlined in Gerlach et al. (2007), a constant gravity-driven flow of 100 mL min^–1^ per channel was maintained throughout all trials using flow meters. Each trial consisted of a 2o-min acclimation period, followed by a 2-min testing period, where the position of the fish, on either the right or left side of the chamber was recoded at 5-sec intervals. Then, there was a 1-min rest period and the water sources were switched from one side to the other, a measure to ensure a side preference was not being displayed. The 2-min acclimation period and 2-min testing period were then repeated. Dye tests were conducted at each water change to ensure that the two flow channels exhibited parallel water flow with no areas of turbulence or eddies.

All trials were conducted on a minimum of 15 newly settled individuals of each species. Each individual was only tested once. Kolmogorov–Smirnov tests were used to compare the proportion of time that individuals spent in the stream of water containing an olfactory cue compared to the proportion of time that individuals spent on one side of the chamber when no cues were present (i.e., offshore water), results from the blank control trial.

### Olfactory choice trials

#### Experiment 1: Olfactory discrimination between water samples from reefs with and without islands

Pairwise choice experiments were conducted to test whether juveniles could discriminate between water collected at reefs surrounding islands and water collected at reefs with no islands (i.e., submerged reefs separated by >10 km from the nearest island). On reefs surrounding islands, water was collected 1 m from the shoreline. On reefs without islands, water was collected from the center of the reef flat at its shallowest point. Water was collected from at least two different islands and nonisland locations for each fish species, to ensure olfactory preferences displayed were a result of general olfactory preferences rather than a response to a specific location. All water collection occurred during the ebb tide.

#### Experiment 2: Olfactory discrimination from water sampled at different distances away from island shoreline

To determine if olfactory cues are used for distinguishing between different habitat locations within reefs that surround islands, juveniles were given pairwise choices between water from three different positions associated with island reefs: (1) beach water (collected within 1 m off the shoreline), (2) reef crest water (collected from near the outer edge of the reef crest), and (3) offshore water (collected 1 km from the reef crest, measured using a global positioning system). All possible combinations of the three water sources were tested against each other.

#### Experiment 3: Olfactory discrimination between water samples treated with terrestrial cues

To test if island-associated species are attracted to the chemical cues of tropical plants, juveniles were given pairwise choices between offshore water treated with terrestrial leaf litter and untreated offshore water. Offshore water was collected 1 km from the reef crest of the island and was chosen as it was assumed to contain less background terrestrial cues than water from near the island. Beach almond (*Terminalia catappa*) was chosen as the terrestrial chemical cue; this species elicited a positive response from *A. percula* (Dixson et al. 2008) and is a common shoreline plant on all offshore islands visited. For each trial, 20 g of leaves, leaf matter was added to offshore water samples (9 L) and allowed to stand for 2 h before experimental use.

## Results

### Association with island reefs

All eight island-associated study species exhibited a strong association with the fringing reefs surrounding islands. For each species, the mean density was significantly higher on reefs surrounding islands than on reefs where no island was present (Table 1, supplementary table 1). All four species of pomacentrids (*P. simsiang, D. prosopotaenia, Dascyllus melanurus* and *A. percula*) were only recorded on reefs that surrounded islands, with no individuals found on reefs where no islands were present. *Dischitodus. melanurus* displayed the strongest island association with a mean density of 42.5 individuals per island reef (*F* = 89.33, *P* < 0.001). Both Chaetodonids showed strong islands associations, with *C. vagabundus* exhibiting five times higher density at island locations opposed to reefs with no islands (*F* = 5.74, *P* < 0.03) and *C. rafflesi* had a mean density of 15.1 individuals on reefs surrounding vegetated islands compared to 0.9 individuals on reefs with no islands present (*F* = 7.65, *P* < 0.01). The labrids surveyed displayed similar levels of association, *H. chloropterus* individual mean density on reefs surrounding islands was three times higher than the individual mean density on reefs with no islands (*F* = 56.40, *P* < 0.001), *H. argus* also showed a higher mean density between the two location types by 3.6 times (*F* = 61.34, *P* < 0.001). As expected, *A. melanopus* did not show an island association, with no significant difference found in density between reefs surrounding islands and reefs where no islands were present (*F* = 1.00, *P* > 0.33)

**Table 1:** Results of island association and pairwise olfactory choice experiments on field-collected juvenile coral reef fish. A nested ANOVA was used to determine if species were significantly associated with island reef habitats over reefs where no islands are present. Choice results include the choices made in experiment 1 comparing water from reefs with and without islands, experiment 2 comparing water from different distances away from islands, experiment 3 comparing water treated with the olfactory cues of rainforest leaves to untreated offshore water. Data are mean percentage of time spent in water flowing from the two sources ± SE. Kolmogorov–Smirnov tests were conducted to determine if preferences are significantly different to the blank trial run for each species. *n*, sample size; *P*, probability of the data given the null hypothesis that there is no choice

Species (mean length, mm)	Island association	Experiment	Water choice 1, mean % time spent ± SE	Water choice 2, mean % time spent ± SE	*n*	*P*
*Cheatodon vagabundus* (23.19 ± 1.36)	Island: *F* = 5.74, *P* < 0.03 reef (island): *F* = 0.70, *P* > 0.60	1	Tuare island 93%± 0.54	South bay reef 7%± 0.54	20	<0.001
			Kimbe island 94%± 0.49	May reef 6%± 0.49	23	<0.001
		2	Beach 91%± 0.35	Offshore 9%± 0.35	25	<0.001
			Reef crest 92%± 0.48	Offshore 8%± 0.48	22	<0.001
			Beach 93%± 0.42	Reef crest 7%± 0.42	25	<0.001
		3	Leaf 86%± 0.56	Offshore 14%± 0.56	16	<0.001
*Cheatodon rafflesii* (21.98 ± 1.03)	Island: *F* = 7.65, *P* < 0.01 reef (island): *F* = 0.89, *P* > 0.49	1	Banban island 94%± 0.41	No name reef 6%± 0.41	21	<0.001
			Kimbe island 95%± 0.19	May reef 5%± 0.19	15	<0.001
		2	Beach 93%± 0.30	Offshore 7%± 0.30	15	<0.001
			Reef crest 89%± 0.35	Offshore 11%± 0.35	15	<0.001
			Beach 93%± 0.42	Reef crest 7%± 0.42	15	<0.001
		3	Leaf 92%±0.40	Offshore 8%±0.40	15	<0.001
*Pomacentrus simsiang* (21.43 ± 1.21)	Island: F = 55.31, *P* < 0.001 reef (island): *F* = 3.12, *P* < 0.04	1	Garove island 95%± 0.38	South bay reef 5%± 0.38	15	<0.001
			Banban island 93%± 0.73	No name reef 7%± 0.73	15	<0.001
		2	Beach 95%± 0.38	Offshore 5%± 0.38	15	<0.001
			Reef crest 87%± 0.53	Offshore 13%± 0.53	15	<0.001
			Beach 91%± 0.37	Reef Crest 9%± 0.37	15	<0.001
		3	Leaf 90%± 0.42	Offshore 10%± 0.42	15	<0.001
*Dischistodus prosopotaenia* (20.56 ± 0.84)	Island: *F* = 22.98, *P* < 0.001 reef (island): *F* = 2.32, *P* > 0.95	1	Banban island 92%± 0.24	No name reef 8%± 0.24	15	<0.001
			Garove island 94%± 0.27	South bay reef 6%± 0.27	15	<0.001
		2	Beach 88%± 0.52	Offshore 12%± 0.52	15	<0.001
			Reef crest 87%± 0.65	Offshore 13%± 0.65	15	<0.001
			Beach 88%± 0.58	Reef Crest 8%± 0.58	15	<0.001
		3	Leaf 79%± 0.46	Offshore 21%± 0.46	15	<0.001
*Dascyllus melanurus* (19.84 ± 0.51)	Island: *F* = 89.33, *P* < 0.001 Reef (Island): *F* = 1.58, *P* > 0.22	1	Banban island 93%± 0.51	No name reef 7%± 0.51	15	<0.001
			Garove island 93%± 0.56	South bay reef 7%± 0.56	15	<0.001
		2	Beach 92%± 0.51	Offshore 8%± 0.51	15	<0.001
			Reef crest 88%± 0.51	Offshore 12%± 0.51	15	<0.001
			Beach 90%± 0.34	Reef crest 10%± 0.34	15	<0.001
		3	Leaf 88%± 0.50	Offshore 12%± 0.50	20	<0.001
*Amphiprion percula* (20.79 ± 0.84)	Island: F = 97.31, *P* < 0.001 reef (island): *F* = 0.83, *P* > 0.52	1	Tuare island 99%± 0.18	South bay reef 1%± 0.18	30	<0.001
			Kimbe island 97%± 0.23	May reef 3%± 0.23	30	<0.001
		2	Beach 97%± 0.26	Offshore 3%± 0.26	30	<0.001
			Reef crest 95%± 0.41	Offshore 5%± 0.41	30	<0.001
			Beach 97%± 0.19	Reef crest 3%± 0.19	30	<0.001
*Halichoeres argus* (23.71 ± 2.41)	Island: *F* = 61.34, *P* < 0.001 reef (island): *F* = 27.08, *P* < 0.001	1	Tuare island 92%± 0.20	South bay reef 8%± 0.20	15	<0.001
			Kimbe island 92%± 0.28	May reef 8%± 0.28	15	<0.001
		2	Beach 96%± 0.36	Offshore 4%± 0.36	15	<0.001
			Reef crest 84%± 0.43	Offshore 16%± 0.43	15	<0.001
			Beach 94%± 0.45	Reef crest 6%± 0.45	15	<0.001
		3	Leaf 94%± 0.23	Offshore 6%± 0.23	15	<0.001
*Halichoeres chloropterus* (19.43 ± 0.78)	Island: *F* = 56.40, *P* < 0.001 reef (island): *F* = 10.39, *P* < 0.001	1	Tuare island 95%± 0.20	South bay reef 5%± 0.20	15	<0.001
			Kimbe island 93%± 0.54	May reef 7%± 0.54	15	<0.001
		2	Beach 96%± 0.35	Offshore 4%± 0.35	15	<0.001
			Reef crest 88%± 0.40	Offshore 12%± 0.40	15	<0.001
			Beach 965 ± 0.40	Reef crest 6%± 0.40	15	<0.001
		3	Leaf 99%± 0.17	Offshore 1%± 0.17	15	<0.001
*Amphiprion melanopus* (21.43 ± 1.07)	Island: *F* = 1.00, *P* > 0.33 reef (island): *F* = 1.00, *P* > 0.43	1	Tuare island 48%± 0.30	South bay reef 52%± 0.30	10	>0.10
			Garove island 51%± 0.49	South bay reef 49%± 0.49	10	>0.10
		2	Beach 86%± 1.18	Offshore 14%± 1.18	10	<0.001
			Reef crest 84%± 1.14	Offshore 16%± 1.14	10	<0.001
			Beach 50%± 0.45	Reef crest 50%± 0.45	10	>0.10
		3	Leaf 48%± 0.39	Offshore 52%± 0.39	10	>0.10

### Olfactory choice trials

#### Experiment 1: Olfactory discrimination between water samples from reefs with and without islands

All island-associated species spent a significantly greater amount of time in the olfactory cues from water collected from reefs surrounding islands over water collected from reefs where no island was present (Table 1). This indicates that these species are all able to discriminate between olfactory cues, as both water sources would contain different olfactory compounds. All species spent a minimum of 92% of their time in the olfactory cues collected from reefs surrounding islands, over the olfactory cues collected from reefs alone. The strongest island preference was shown by *A. percula*, spending between 97% and 99% of the time in the island water, while both *H. argus* and *D. prosopotaenia* exhibited the weakest preference for island water, still spending 92% of their time in the island cue. The nonisland-associated species, *A. melanopus* spent approximately equal amount of time in the water collected from the island location compared to the water collected from the reef with no island, for either set of locations tested (*P* > 0.10).

#### Experiment 2: Olfactory discrimination from water sampled at different distances away from island shoreline

All nine species were able to discriminate between water collected at different distances from the reef. Juveniles spent a disproportionate amount of time (>84%) in water collected from both the beach and reef crest location over the water collected 1-km offshore (Table 1). Island-associated recruits were also able to discriminate between the reef crest water and beach water, showing a strong preference for the beach water, *A. melanopus* however did not display a preference for beach water over reef crest water with recruits spending equal time in either water stream (Table 1). Beach water was preferred by island-associated species, when compared against either reef crest water or offshore water, with juveniles spending greater than 90% of their time in the beach water.

#### Experiment 3: Olfactory discrimination between water samples treated with terrestrial cues

All eight island-associated study species exhibited significant discrimination between offshore water treated with leaves of a common rainforest plant, *T. catappa* versus untreated offshore water; responding positively to the olfactory cue from terrestrial leaf litter. Each species spent >79% (and up to 99%) of observations in offshore water treated with leaf litter compared to untreated offshore water (Table 1). The weakest response (79% of time in offshore water treated with leaf litter) was found in *D. prosopotaenia*, and the strongest response (99%) was found in *H. chloropterus*. *Amphiprion melanopus* did not display a preference for the leaf litter cue. Importantly, the leaf litter cue was neither avoided nor preferred by this species (Table 1).

## Discussion

All eight island-associated fish species had significantly higher densities on reefs surrounding islands compared to reefs with no islands present. Some species tested were found exclusively at the island reef location, therefore species used in this study are strongly associated to the island location and may require a mechanism for island/reef identification. All species could discriminate between water from reefs surrounding islands and nonisland reefs; indicating that they are able to use olfactory cues to identify island reefs. Each species was also able to discriminate between water taken at different distances from an island, with the exception of *A. melanopus* species preferred the beach location, presumably due to a higher concentration of distinct olfactory cues arising from island itself. All eight island-associated species were also capable of detecting a difference between offshore water treated with terrestrial leaf litter and untreated offshore water. In all trials, island-associated fish showed a strong preference for water containing terrestrial cues, or the greatest concentration of terrestrial cues. One of the striking features of our results is the strength and consistency of the preferences within and among species. In Experiment 1, all island-associated juveniles, regardless of species, spent over 92% of their time in the water from reefs surrounding islands. A clear preference was also shown by all island-associated species for beach water over offshore water as well as reef crest water compared to offshore water. Variation in cue preference within species was extremely low. Perhaps most remarkable, is the strength of attraction to the olfactory cues produced by terrestrial leaf litter. In this experiment, the strength of the preference varied the most among species, with preferences ranging from 79 to 99%, but one of the species, *H. chloropterus*, displayed the strongest preference (99%) seen among all three experiments.

*Amphiprion melanopus* displayed a significant reaction to the olfactory cues of the beach water and the reef crest when presented against offshore water, however showed no preference for beach water when tested against reef crest water. This indicates that the initial preference for beach and reef crest water was because it is seen as suitable reef habitat opposed to no olfactory cue found in offshore water, however when beach water was presented against reef crest water, both containing reef odor, no choice was made. *Amphiprion melanopus* also displayed no preference for or against the chemical cues from terrestrial leaves, indicating that this is not a utilized settlement cue for this generalist species. It is important to note that the island cues were not avoided by *A. melanopus*, indicating that reefs, which surround islands are not preferred over other reefs but will be utilized as habitat and recognized through reef cues rather than terrestrial cues.

Our experiments were conducted on newly settled fish, which were not naive to the island cues, therefore, it is possible that a preferences for learned cues might have contributed to the observed preferences. However, previous research with the clownfish *A. percula* has demonstrated that preferences for cues from tropical island plants are innate (Dixson et al. 2008). Furthermore, the nonisland-associated species, *A. melanopus*, did not exhibit a preference for island water, even when collected from island locations, which is consistent with an innate response rather than a learned response.

Reef fish are capable of responding to chemical cues from anemones (Elliott et al. 1995; Arvedlund and Nielsen 1996; Arvedlund et al. 1999), conspecifics (Sweatman 1988; Booth 1992; Lecchini et al. 2005), and live coral (Lecchini et al. 2005; Ben–Tzvi et al. 2010). However, the chemical cues from these sources may be of greatest importance once the larvae are already within the reef matrix. The chemical signals produced by terrestrial leaf litter may be detected over a greater distance, allowing island-associated fish to use this cue before coming into contact with the reef itself. The strength of attraction supports the island mass effect (sensu Gilmartin and Revelante 1974), which predicts that the export of leaf litter causes island habitats to be a bigger target than their actual size for larvae that respond to these terrestrial cues. The use of olfactory cues in habitat selection has also been shown among seagrass-associated fishes (Nagelkerken et al. 2002). For example, the Spangled emperor, *Lethrinus nebulosus*, uses olfactory cues produced by seagrasses to distinguish this habitat from rubble (Arvedlund and Takemura 2006). Results from hydrodynamic modeling have also shown that collective chemical cues from habitats extend significant distances into oceanic environments. For example, the lagoons of coral reefs contain high concentrations of mucous and dissolved organic compounds from corals and other associated organisms (Davies and Hughes 1983). This material can be transported out of the lagoon in turbid plumes that are tens of meters to kilometers long (Booth et al. 2000). Water from the continental shelf of the Great Barrier Reef and its associated lagoons can generate a chemical gradient detectable by larvae in the Coral Sea (Wolanski 1994). While identifying specific chemical cues remains difficult, research has shown that chemical cues carrying useful information are able to disperse great distances from their source with the potential to be used by navigating larvae.

Conservation plans are typically designed to encompass single ecosystems. However, as this study suggests, ecosystems do not function independently from one another, further supporting the need for integrated management policies between the terrestrial and the marine environments. Although the earlier work on *A. percula* (Dixson et al. 2008) has already suggested that terrestrial cues are important, an even stronger link can now be made between coral reefs and rainforest habitat with the addition of seven other island-associated species using the same terrestrial signals. Connectivity among ecosystems has been demonstrated in terms of transfer of energy and nutrients from one ecosystem to another, as well as in terms of life-history movements and movements of adults (reviewed by Beger et al. 2010). For example, extensive mangrove habitat in the Caribbean has been shown to positively influence the biomass of fishes on coral reefs (Mumby et al. 2004), and is also important as a nursery habitat for a number of commercially important coral reef species (Nagelkerken et al. 2000, 2002, 2008). Some coastal species such as the Coconut crab (*Birgus latro*) have a pelagic larval stage, which uses the oceanic environment for biological dispersal and must locate suitable coastal habitat at the conclusion of their larval stage (Lavery et al. 1996). Our study demonstrates a unique terrestrial–marine link in a group of coral reef species for which such a link would not be expected.

Coastal environments, including vegetated islands, are often the focal point for human activity whether it is recreational or agricultural; both resulting in significant loss of native rainforest vegetation. This study has demonstrated the importance of native vegetation through chemical cues in the recognition of appropriate reef habitat by a number of coral reef fish species. Removal of native cues could potentially affect patterns of connectivity if larvae are relying on the use of terrestrial cues to locate reef habitat. Although formulating management plans that include multiple ecosystems adds complexity and cost to an already complicated process, the connectivity between different ecosystems cannot be ignored.

## Conclusion

This study suggests that the rainforest vegetation is an important chemical cue for location of suitable settlement sites among island-associated coral reef fish. There is still significant further research required, including identifying the chemical compounds involved and at what spatial scales they are detected. Nonetheless, our results demonstrate the broad importance of the link between the coral reef and rainforest ecosystem.

## Supporting Information

**Table S1.** Results from a nested analysis of variance (ANOVA), used to compare the log-transformed data of fish densities between reefs with it and without islands. Bold text indicates no island association.
