# Correction to “Terrestrial Chemical Cues Help Coral Reef Fish Larvae Locate Settlement Habitat Surrounding Islands”

**DOI:** 10.1002/ece3.73001

**Published:** 2026-02-11

Dixson, D. L., G. P. Jones, P. L. Munday, et al., “Terrestrial Chemical Cues Help Coral Reef Fish Larvae Locate Settlement Habitat Surrounding Islands.” *Ecology and Evolution* 1, no. 4 (2011): 586–595. https://doi.org/10.1002/ece3.53.

A mistake was identified in the calculation of the standard errors in Table 1. The errors do not affect the overall results or conclusions. The corrected version of the table is below:

**TABLE 1:** Results of island association and pairwise olfactory choice experiments on field‐collected juvenile coral reef fish. A nested ANOVA was used to determine if species were significantly associated with island reef habitats over reefs where no islands are present. Choice results include the choices made in experiment 1 comparing water from reefs with and without islands, experiment 2 comparing water from different distances away from islands, experiment 3 comparing water treated with the olfactory cues of rainforest leaves to untreated offshore water. Data are mean percentage of time spent in water flowing from the two sources ±SE. Kolmogorov‐Smirnov tests were conducted to determine if preferences are significantly different to the blank trial run for each species. *n*, sample size; *p*, probability of the data given the null hypothesis that there is no choice.

Species (mean length, mm)	Island association	Experiment	Water choice 1, mean % time spent ±SE	Water choice 2, mean % time spent ±SE	*n*	*p*
*Cheatodon vagabundus* (23.19 ±1.36)	Island: *F* = 5.74, *p* < 0.03 Reef (Island): *F* = 0.70, *p* > 0.60	1	Tuare Island 93% ±0.66	South Bay Reef 7% ±0.66	20	< 0.001
			Kimbe Island 94% ±0.63	May Reef 6% ±0.63	23	< 0.001
		2	Beach 91% ±0.48	Offshore 9% ±0.48	25	< 0.001
			Reef Crest 92% ±0.69	Offshore 8% ±0.69	22	< 0.001
			Beach 93% ±0.55	Reef Crest 7% ±0.55	25	< 0.001
		3	Leaf 86% ±1.30	Offshore 14% ±1.30	16	< 0.001
*Cheatodon rafflesii* (21.98 ±1.03)	Island: *F* = 7.65, *p* < 0.01 Reef (Island): *F* = 0.89, *p* > 0.49	1	Banban Island 94% ±1.17	No Name Reef 6% ±1.17	21	< 0.001
			Kimbe Island 95% ±0.40	May Reef 5% ±0.40	15	< 0.001
		2	Beach 93% ±0.68	Offshore 7% ±0.68	20	< 0.001
			Reef Crest 89% ±0.83	Offshore 11% ±0.83	20	< 0.001
			Beach 93% ±0.97	Reef Crest 7% ±0.97	20	< 0.001
		3	Leaf 92% ±0.91	Offshore 8% ±0.91	15	< 0.001
*Pomacentrus simsiang* (21.43 ±1.21)	Island: *F* = 55.31, *p* < 0.001 Reef (Island): *F* = 3.12, *p* < 0.04	1	Garove Island 95% ±0.93	South Bay Reef 5% ±0.93	15	< 0.001
			Banban Island 93% ±0.78	No Name Reef 7% ±0.78	15	< 0.001
		2	Beach 95% ±1.16	Offshore 5% ±1.16	15	< 0.001
			Reef Crest 87% ±1.07	Offshore 13% ±1.07	15	< 0.001
			Beach 91% ±0.74	Reef Crest 9% ±0.74	15	< 0.001
		3	Leaf 90% ±1.09	Offshore 10% ±1.09	15	< 0.001
*Dischistodus prosopotaenia* (20.56 ±0.84)	Island: *F* = 22.98, *p* < 0.001 Reef (Island): *F* = 2.32, *p* > 0.95	1	Banban Island 92% ±1.07	No Name Reef 8% ±1.07	15	< 0.001
			Garove Island 94% 1.07	South Bay Reef 6% 1.07	15	< 0.001
		2	Beach 88% ±1.08	Offshore 12% ±1.08	20	< 0.001
			Reef Crest 87% ±1.36	Offshore 13% ±1.36	20	< 0.001
			Beach 88% ±1.21	Reef Crest 12% ±1.21	20	< 0.001
		3	Leaf 79% ±1.07	Offshore 21% ±1.07	15	< 0.001
*Dascyllus melanurus* (19.84 ±0.51)	Island: *F* = 89.33, *p* < 0.001 Reef (Island): *F* = 1.58, *p* > 0.22	1	Banban Island 93% ±0.72	No Name Reef 7% ±0.72	15	< 0.001
			Garove Island 93% ±1.53	South Bay Reef 7% ±1.53	15	< 0.001
		2	Beach 92% ±1.06	Offshore 8% ±1.06	20	< 0.001
			Reef Crest 88% ±1.06	Offshore 12% ±1.06	20	< 0.001
			Beach 90% ±0.70	Reef Crest 10% ±0.70	20	< 0.001
		3	Leaf 88% ±1.39	Offshore 12% ±1.39	15	< 0.001
*Amphiprion percula* (20.79 ±0.84)	Island: *F* = 97.31, *p* < 0.001 Reef (Island): *F* = 0.83, *p* > 0.52	1	Tuare Island 99% ±0.30	South Bay Reef 1% ±0.30	30	< 0.001
			Kimbe Island 97% ±0.59	May Reef 3% ±0.59	30	< 0.001
		2	Beach 97% ±0.76	Offshore 3% ±0.76	30	< 0.001
			Reef Crest 95% ±0.60	Offshore 5% ±0.60	30	< 0.001
			Beach 97% ±0.73	Reef Crest 3% ±0.73	30	< 0.001
*Halichoeres argus* (23.71 ±2.41)	Island: *F* = 61.34, *p* < 0.001 Reef (Island): *F* = 27.08, *p* < 0.001	1	Tuare Island 92% ±0.56	South Bay Reef 8% ±0.56	15	< 0.001
			Kimbe Island 92% ±1.09	May Reef 8% ±1.09	15	< 0.001
		2	Beach 96% ±0.76	Offshore 4% ±0.76	15	< 0.001
			Reef Crest 84% ±0.89	Offshore 16% ±0.89	15	< 0.001
			Beach 94% ±0.94	Reef Crest 6% ±0.94	15	< 0.001
		3	Leaf 94% ±0.95	Offshore 6% ±0.95	15	< 0.001
*Halichoeres chloropterus* (19.43 ±0.78)	Island: *F* = 56.40, *p* < 0.001 Reef (Island): *F* = 10.39, *p* < 0.001	1	Tuare Island 95% ±0.74	South Bay Reef 5% ±0.74	15	< 0.001
			Kimbe Island 93% ±0.75	May Reef 7% ±0.75	15	< 0.001
		2	Beach 96% ±0.73	Offshore 4% ±0.73	15	< 0.001
			Reef Crest 88% ±0.83	Offshore 12% ±0.83	15	< 0.001
			Beach 96% ±0.83	Reef Crest 4% ±0.83	15	< 0.001
		3	Leaf 99% ±0.40	Offshore 1% ±0.40	15	< 0.001
*Amphiprion melanopus* (21.43 ±1.07)	Island: *F* = 1.00, *p* > 0.33 Reef (Island): *F* = 1.00, *p* > 0.43	1	Tuare Island 48% ± 0.79	South Bay Reef 52% ±0.79	10	> 0.10
			Garove Island 51% ±1.25	South Bay Reef 49% ±1.25	10	> 0.10
		2	Beach 86% ±2.45	Offshore 14% ±2.45	10	< 0.001
			Reef Crest 84% ±2.37	Offshore 16% ±2.37	10	< 0.001
			Beach 50% ±0.93	Reef Crest 50% ±0.93	10	> 0.10
		3	Leaf 48% ±1.28	Offshore 52% ±1.28	10	> 0.10

The authors apologize for this error.

