# Supplementary material for: Terrestrial chemical cues help coral reef fish larvae locate settlement habitat surrounding islands
Source: Ecol Evol. 2011 Dec;1(4):586–95. doi: 10.1002/ece3.53 (PMC3287328; doi:10.1002/ece3.53)
Supplement: Supplementary file 1 [file ece30001-0586-SD1.doc]

Supplementary Materials

| **Species** | **Factor** | **df** | **SS** | **MS** | **F** | **p** |
| --- | --- | --- | --- | --- | --- | --- |
| *Cheatodon vagabundus* | Island/ Non-island | 1 | 0.395057 | 0.395057 | 7.65022 | 0.012733 |
|  | Reef(Island/ Non-island) | 4 | 0.184027 | 0.046007 | 0.89092 | 0.489428 |
|  | Error | 18 | 0.929518 | 0.051640 |  | |
| *Cheatodon rafflesii* | Island/ Non-island | 1 | 1.392793 | 1.392793 | 18.37387 | 0.000444 |
|  | Reef(Island/ Non-island) | 4 | 0.177794 | 0.044448 | 0.58637 | 0.676625 |
|  | Error | 18 | 1.364452 | 0.075803 |  | |
| *Pomacentrus simsiang* | Island/ Non-island | 1 | 4.178314 | 4.178314 | 53.30742 | 0.000001 |
|  | Reef(Island/ Non-island) | 4 | 0.977149 | 0.244287 | 3.11665 | 0.041071 |
|  | Error | 18 | 1.410867 | 0.078381 |  | |
| *Dischistodus prosopotaenia* | Island/ Non-island | 1 | 2.531002 | 2.531002 | 22.97888 | 0.000145 |
|  | Reef(Island/ Non-island) | 4 | 1.023606 | 0.255901 | 2.32332 | 0.095930 |
|  | Error | 18 | 1.982605 | 0.110145 |  | |
| *Dascyllus melanurus* | Island/ Non-island | 1 | 10.33541 | 10.33541 | 89.3280 | 0.000000 |
|  | Reef(Island/ Non-island) | 4 | 0.73070 | 0.18268 | 1.5788 | 0.222818 |
|  | Error | 18 | 2.80263 | 0.11570 |  | |
| *Amphiprion percula* | Island/ Non-island | 1 | 5.799495 | 5.799495 | 97.30981 | 0.000000 |
|  | Reef(Island/ Non-island) | 4 | 0.196879 | 0.049220 | 0.82586 | 0.525797 |
|  | Error | 18 | 1.072766 | 0.059598 |  | |
| *Halichoeres argus* | Island/ Non-island | 1 | 2.20609 | 2.20609 | 61.3392 | 0.000000 |
|  | Reef(Island/ Non-island) | 4 | 3.89520 | 0.97380 | 27.0720 | 0.000000 |
|  | Error | 18 | 0.64738 | 0.03597 |  | |
| *Halichoeres chloropterus* | Island/ Non-island | 1 | 2.068494 | 2.068494 | 56.0052 | 0.000001 |
|  | Reef(Island/ Non-island) | 4 | 1.524806 | 0.381201 | 10.39402 | 0.000153 |
|  | Error | 18 | 0.660152 | 0.036675 |  | |
| ***Amphiprion melanopus*** | **Island/ Non-island** | **1** | **0.009485** | **0.009485** | **1.000000** | **0.330565** |
|  | **Reef(Island/ Non-island)** | **4** | **0.037941** | **0.009485** | **1.000000** | **0.433165** |
|  | **Error** | **18** | **0.170734** | **0.009485** |  | |
